# Supplementary material for: Transcriptomic Analysis of the Anticancer Effects of Annatto Tocotrienol, Delta-Tocotrienol and Gamma-Tocotrienol on Chondrosarcoma Cells
Source: Nutrients. 2022 Oct 13;14(20):4277. doi: 10.3390/nu14204277 (PMC9611384; doi:10.3390/nu14204277)
Supplement: Supplementary file 1 [file nutrients-14-04277-s001.zip › S1 - The primer sequences used in this study.pdf]

Supplementary Table S1: The primer sequences used in this study

**Gene name**	**Sequences**	
GAPDH	Forward	5’- GTCAGTGGTGGACCTGACCT-3’
	Reverse	5’-ACCTGGTGCTCAGTGTAGCC-3’
ADAMTSL1	Forward	5’- TTAAGCTCATCGGAGGCAAC-3’
	Reverse	5’- AGATCCCGTTCTGGTGTTTG-3’
HMGCS1	Forward	5’- TGTGGAGAAGGCATTTATGAAG-3’
	Reverse	5’- AGAACAGATGCAAGGGAACC-3’
KLHDC7B	Forward	5’- CTGCACCATGCACAACTACC-3’
	Reverse	5’- GAACCTGGCTCCAGATGTTG-3’
ARMCX3	Forward	5’- TCGCTTGAACTCATCTGTGC-3’
	Reverse	5’- TTTCCCGCTGAAAATAAACG-3’
