# Supplementary material for: Transcriptomic Analysis of the Anticancer Effects of Annatto Tocotrienol, Delta-Tocotrienol and Gamma-Tocotrienol on Chondrosarcoma Cells
Source: Nutrients. 2022 Oct 13;14(20):4277. doi: 10.3390/nu14204277 (PMC9611384; doi:10.3390/nu14204277)
Supplement: Supplementary file 1 [file nutrients-14-04277-s001.zip › S2 - The purity, quality and concentration of isolated RNAs.pdf]

Supplementary Table S2: The purity, quality and concentration of isolated RNAs

Groups	Sample ID	RNA concentration (ng/µL)	260/280 ratio	RIN
VC	V1	309.5	2.05	9.5
	V2	278.4	2.06	9.8
	V3	295.5	2.05	9.5
AnTT	A1	164.3	2.05	9.2
	A2	166	2.07	9.6
	A3	135.5	2.05	8.1
γ-T3	G1	115.6	2.06	9.3
	G2	111.3	2.06	8.9
	G3	123.9	2.05	8.5
δ-T3	D1	116.2	2.06	9.1
	D2	120.1	2.07	9.1
	D3	134.3	2.06	9.1

Abbreviations: 260/280 ratio, the ratio of absorbance at 260 nm and 280 nm, AnTT, annatto tocotrienol, RIN, RNA integrity number, δ-T3, δ-tocotrienol, γ-T3, γ-tocotrienol, VC, vehicle control
