# Supplementary material for: Transcriptomic Analysis of the Anticancer Effects of Annatto Tocotrienol, Delta-Tocotrienol and Gamma-Tocotrienol on Chondrosarcoma Cells
Source: Nutrients. 2022 Oct 13;14(20):4277. doi: 10.3390/nu14204277 (PMC9611384; doi:10.3390/nu14204277)
Supplement: Supplementary file 1 [file nutrients-14-04277-s001.zip › S3 - Summary of read mapping quality.pdf]

Supplementary Table S3: Summary of read mapping quality

Groups	Sample ID	Total reads	Trimmed reads	Low quality (Q<20)	Clean reads	Aligned and mapped reads
VC	V1	15,350,229	4,259,523	85,114 (0.55%)	15,265,115 (99.44%)	12,941,555 (84.78%)
	V2	12,834,646	3,754,070	77,022 (0.60%)	12,757,624 (99.40%)	10,702,791 (83.89%)
	V3	15,619,857	4,599,524	82,495 (0.53%)	15,537,362 (99.47%)	13,208,234 (85.01%)
AnTT	A1	14,108,686	4,261,142	86,856 (0.62%)	14,021,830 (99.38%)	11,519,918 (82.16%)
	A2	15,327,571	4,577,364	90,251 (0.59%)	15,237,320 (99.41%)	12,710,520 (83.42%)
	A3	15,327,632	4,752,035	85,222 (0.56%)	15,242,410 (99.44%)	12,571,898 (82.48%)
γ-T3	G1	16,361,773	4,940,168	122,143 (0.75%)	16,239,630 (99.25%)	13,088,726 (80.30%)
	G2	14,836,309	4,325,261	102,929 (0.69%)	14,733,380 (99.31%)	12,082,682 (81.47%)
	G3	15,396,105	4,260,140	90,630 (0.59%)	15,305,475 (99.41%)	12,707,032 (82.08%)
δ-T3	D1	13,273,092	4,154,452	78,506 (0.59%)	13,194,586 (99.41%)	10,595,045 (80.60%)
	D2	13,481,565	4,067,438	76,614 (0.57%)	13,404,951 (99.43%)	10,921,551 (82.01%)
	D3	13,251,041	3,521,157	83,689 (0.63%)	13,167,352 (99.37%)	10,807,338 (83.02%)

Abbreviations: AnTT, annatto tocotrienol, RIN, RNA integrity number, δ-T3, δ-tocotrienol, γ-T3, γ-tocotrienol, VC, vehicle control
